# Serological survey of antibodies to *Toxoplasma gondii* and *Coxiella burnetii* in rodents in north-western African islands (Canary Islands and Cape Verde)

**DOI:** 10.4102/ojvr.v82i1.899

**Published:** 2015-05-29

**Authors:** Pilar Foronda, Josué Plata-Luis, Borja del Castillo-Figueruelo, Ángela Fernández-Álvarez, Aarón Martín-Alonso, Carlos Feliu, Marilena D. Cabral, Basilio Valladares

**Affiliations:** 1 University Institute of Tropical Diseases and Public Health of the Canary Islands, University of La Laguna, Spain; 2 Laboratory of Parasitology, University of Barcelona, Spain; 3 Department of Science and Technology, University of Cape Verde, Cape Verde

## Abstract

*Coxiella burnetii* and *Toxoplasma gondii* are intracellular parasites that cause important reproductive disorders in animals and humans worldwide, resulting in high economic losses. The aim of the present study was to analyse the possible role of peridomestic small mammals in the maintenance and transmission of *C. burnetii* and *T. gondii* in the north-western African archipelagos of the Canary Islands and Cape Verde, where these species are commonly found affecting humans and farm animals. Between 2009 and 2013, 108 black rats (*Rattus rattus*) and 77 mice (*Mus musculus*) were analysed for the presence of *Coxiella* and *Toxoplasma* antibodies by enzyme-linked immunosorbent assay (ELISA) and indirect immunofluorescence (IFA), respectively. Our results showed a wide distribution of *C. burnetii* and *T. gondii*, except for *T. gondii* in Cape Verde, in both rodent species. The overall seroprevalence of *C. burnetii* antibodies was 12.4%; 21.1% for Cape Verde and 10.2% for the Canary Islands. With respect to *T. gondii*, seropositive rodents were only observed in the Canary Islands, with an overall seroprevalence of 15%. Considering the fact that both pathogens can infect a large range of hosts, including livestock and humans, the results are of public health and veterinary importance and could be used by governmental entities to manage risk factors and to prevent future cases of Q fever and toxoplasmosis.

## Introduction

*Coxiella burnetii* and *Toxoplasma gondii* are intracellular pathogens with worldwide distribution (Angelakis & Raoult 2010; Dubey 2010). Q fever is caused by *C. burnetii* and has been found in a large range of domestic and wild animals including mammals, birds, reptiles and arthropods (Angelakis & Raoult 2010). Toxoplasmosis is caused by *T. gondii* and can be found in all warm-blooded animals, including terrestrial and marine mammals and birds (Jenkins *et al.* 2013; Pan *et al.* 2012; Wendte, Gibson & Grigg 2011).

Both pathogens cause high economic losses in livestock as a result of reproductive disorders (Arricau-Bouvery & Rodolakis 2005; Guatteo *et al.* 2011; Hill, Chirukandoth & Dubey 2005). In humans, most infections remain asymptomatic; however, Q fever and toxoplasmosis can be dangerous to pregnant women and immunosuppressed patients (Angelakis & Raoult 2010; Desmonts & Couvreur 1974; Dubey & Beattie 1988).

The main source of human infection with Q fever is livestock. Pets can also transmit this disease to humans (Arricau-Bouvery & Rodolakis 2005) and oral transmission by ingesting dairy products has been also reported (Fishbein & Raoult 1992). In the case of toxoplasmosis, the main source of infection is the ingestion of raw or insufficiently cooked meat with tissue cysts, oocysts in water or raw food and via vertical transmission (Cook *et al.* 2000).

Rodents have been found to be a source of livestock infection with several pathogens, including *T. gondii* (Kijlstra *et al.* 2008) and *C. burnetii* (Reusken *et al.* 2011). Therefore, and considering the fact that both *T. gondii* and *C. burnetii* are endemic in the Canary Islands (Bolaños *et al.* 2003; Rodríguez *et al.* 2010; Rodríguez-Ponce, Molina & Hernández 1995; Solá-Graffigna 1997; Velasco 2010) and the lack of information on these species for Cape Verde, the aim of the present work was to contribute to the understanding of the epidemiology of the two pathogens in these north-western African archipelagos. To this end, a serological study to detect *T. gondii* and *C. burnetii* antibodies in peridomestic rodents from the Canary Islands and Cape Verde was carried out.

## Materials and methods

### Biological samples and study area

The study was carried out in five of the seven Canary Islands (Spain), namely Tenerife, El Hierro, Gran Canaria, Lanzarote and Fuerteventura, and one island belonging to the Cape Verde Republic, namely Santiago. Both archipelagos are located near the north-west coast of Africa. The Canary Islands are located 100 km off the coast of Morocco, between 27°37′N and 29°24′N and 13°23′W and 18°8′W. Cape Verde is located 550 km off the coast of Senegal, between 17°16′N and 14°44′N and 22°37′W and 25°24′W. Between 2009 and 2013, 185 rodents from the Canaries and Cape Verde were captured randomly, comprising 108 black rats (*Rattus rattus*) (Linnaeus, 1758) and 77 mice (*Mus musculus*) (Linnaeus, 1758). The sample size was determined according to the variables host species and island. The sampled areas were determined at random: Orgaos, Praia and Säo Domingos in Santiago, San Cristóbal de La Laguna in Tenerife, Frontera in El Hierro, Telde, Santa Brígida and San Mateo in Gran Canaria and the whole islands of Fuerteventura and Lanzarote. The animals were captured alive using Sherman traps in suburban-rural areas. The traps were set in the afternoon and the evening and checked soon after sunrise. Animals were taken to the University Institute of Tropical Diseases and Public Health of the Canary Islands or to laboratories belonging to Cape Verde University.

The animals were euthanised by cervical dislocation or by carbon dioxide inhalation and bled by cardiac puncture. Blood samples were centrifuged and serum was removed and stored in a 1:1 glycerol solution at -20 °C until analysed.

### Enzyme-linked immunosorbent assay

An ELISA kit was used (*Coxiella burnetii* ELISA kit, Bio-X Diagnostics, Belgium); however, anti-rat antibody peroxidase conjugate and the anti-mouse antibody peroxidase conjugate (Sigma-Aldrich, USA) were used with the serum samples instead of the kit’s anti-goat antibody, which was used in controls. The sera and controls were used at 1:100 dilutions. The plates were read spectrophotometrically at 450 nm with a microplate reader (Model 680, Bio-Rad Laboratories, USA). The cut-off value was determined in accordance with the manufacturer’s instructions.

### Indirect immunofluorescence

An IFA kit was used (Toxo-Spot IFI, Biomerieux, France) in order to detect antibodies against *T. gondii*. The serum samples were diluted at 1:40 and 1:80 in phosphate buffered saline (PBS) and the conjugate was made as follows: 400 µL PBS, 10 µL Evans blue and 1 µL goat anti-rat or goat anti-mouse antibodies (Thermo Scientific, USA), both labelled with fluorescein. For each slide, one negative and one positive control were included and the results were determined in accordance with these controls. A positive sample was used as positive control and PBS was used as negative control.

### Statistical analysis

Data analysis was performed with statistical software SPSS 20.0 (IBM, USA). Logistic regression could not be carried out because of the limited number of outcome events. Instead, chi-square contingency tables were used to examine the relationships between rodent species and island studied and the proportion of rodents harbouring antibodies against *C. burnetii* or *T. gondii*. Fisher’s exact test was used if expected cell counts were < 5. A probability value < 0.05 was considered statistically significant.

## Ethical considerations

All the animal procedures were performed according to the principles of animal welfare in experimental science (Spanish Government 2007, 2013). Animal trapping and its use were approved by the environmental agencies of the relevant governmental entities, in accordance with Act 151/2001, 33/2003, 42/2007 and 4/2010.

## Results

*Coxiella burnetii* and* T. gondii* antibodies were found in both species of rodents analysed, with overall prevalences of 12.4% (CI 95% 7.7–17.2) and 11.9% (CI 95% 7.2–16.6), respectively. *Coxiella burnetii* was present in the two archipelagos studied; *T. gondii* was not found in Cape Verde (Table 1).

Rodents carrying antibodies against *C. burnetii* were found in all the islands analysed except for Tenerife, where antibodies were not found in either host. *C. burnetii* showed a higher seroprevalence in Cape Verde (21.1%, CI 95% 8.1–34.0) than in the Canary Islands (10.2%, CI 95% 5.3–15.1) but without significant differences. Significant differences in the proportion of rodents harbouring antibodies against both *C. burnetii* and *T. gondii* were found amongst the different islands included in the study (*p* < 0.01, Chi-square contingency table). When data from different hosts were compared, significant differences were observed for *C. burnetii* between *M. musculus* (16.9%, CI 95% 7.81–26.0) and *R. rattus* (4.9%, CI 95% 0.2–9.5) (*p* < 0.05) collected from the Canary Islands. However, differences between hosts were not observed when these islands were compared pairwise*.*

**TABLE 1:** Seroprevalence of antibodies against *Coxiella burnetii* and *Toxoplasma gondii* in rodents in the Canary Islands and Cape Verde.

Area and host species	N	C. burnetii	(+) %	T. gondii	(+) %
Canary Islands	147	15	10.2	22	15
*M. musculus*	65	11	16.9	8	12.3
*R. rattus*	82	4	4.9	14	17.1
Tenerife	43	0	0	12	27.9
*M. musculus*	7	0	0	0	0
*R. rattus*	36	0	0	12	33.3
El Hierro	38	6	15.8	1	2.6
*M. musculus*	25	4	16	1	4
*R. rattus*	13	2	15.4	0	0
Gran Canaria	22	1	4.5	1	4.5
*M. musculus*	11	0	0	0	0
*R. rattus*	11	1	9.1	1	9.1
Fuerteventura	22	5	22.7	7	31.8
*M. musculus*	11	5	45.5	6	54.5
*R. rattus*	11	0	0	1	9.1
Lanzarote	22	3	13.6	1	4.5
*M. musculus*	11	2	18.2	1	9.1
*R. rattus*	11	1	9.1	0	0
Cape Verde (Santiago)	38	8	21.1	0	0
*M. musculus*	12	0	-	0	0
*R. rattus*	26	8	30.8	0	0
**Total**	**185**	**23**	**12.4**	**185**	**11.9**
***M. musculus***	**77**	**11**	**14.3**	**8**	**10.4**
***R. rattus***	**108**	**12**	**11.1**	**14**	**12.9**

*N*, sample size; +, number of seropositive rodents; %, percentage of seropositive rodents; *M. musculus: Mus musculus; R. rattus, Rattus rattus; C. burnetii, Coxiella burnetii; T. gondii, Toxoplasma gondii.*

The overall prevalence for *T. gondii* in the Canary Islands was 15% (CI 95% 9.2–20.7); 17.1% (CI 95% 8.9–25.2) for *R. rattus* and 12.3% (CI 95% 4.3–20.3) for *M. musculus*. No significant differences between hosts were found, even when the islands were analysed separately. Comparing the seroprevalence of *T. gondii* between different islands, there was a significant difference for *M. musculus* between Fuerteventura (54.5%, CI 95% 25.1–84.0) and El Hierro (4%, CI 95% 0–11.7) islands (*p* < 0.001, Fisher’s exact test). No positive results were found in rodents captured in Cape Verde.

## Discussion

This study reveals the wide distribution of *T. gondii* and *C. burnetii* in rodents in the Canary Islands, as antibodies against both pathogens were found in all the islands analysed, except for *C. burnetii* in Tenerife. Furthermore, this study also indicates the role of black rats (*R. rattus*) as reservoirs of *T. gondii* in Santiago, as antibodies against this parasite were found in almost one-third of the individuals analysed.

To the authors’ knowledge, there are no previous studies of *C. burnetii* or *T. gondii* in rodents from oceanic islands. When comparing the results with previous studies carried out in continental areas, the overall prevalence of *T. gondii* obtained in the present study (12.4%) is lower than that observed in Iran (24.41%) (Mosallanejad *et al.* 2012) but higher than that found in France (5%) (Gotteland *et al.* 2013) or Brazil (5.7%) (Siqueira *et al.* 2013). The overall prevalence of *C. burnetii* was 11.9%, similar to that obtained in the Netherlands (7.1%) (Reusken *et al.* 2011).

In the Canary Islands, previous studies involving small ruminant livestock (Rodríguez *et al.* 2010; Velasco 2010) and humans (Bolaños *et al.* 2003) have indicated that *C. burnetii* is endemic and *T. gondii* is widely distributed in the archipelago (Rodríguez-Ponce *et al.* 1995; Solá-Graffigna 1997). In the case of Cape Verde, to the authors’ knowledge, there are neither available data for livestock census nor data about *C. burnetii* and *T. gondii*. However, based on personal observations during this study, the main livestock animals in Cape Verde seem to be goats, as in the Canary Islands (Gobierno de Canarias 2012). The same occurs in several African continental countries, such as Uganda, where goats are not only important to the local economy but also serve as a major source of protein (Bisson *et al.* 2000).

Considering the fact that *C. burnetii* and *T. gondii* can be transmitted from rodents to livestock (Kijlstra *et al.* 2008; Reusken *et al.* 2011), the presence of these pathogens in rodents from the Canary Islands and Cape Verde may have influenced the productivity of small ruminant flocks, which depends greatly on their reproductive efficiency (Arricau-Bouvery & Rodolakis 2005; Guatteo *et al.* 2011; Hill *et al.* 2005). However, the results of this study are not only important from an economic point of view; these pathogens are also relevant to public health. Both toxoplasmosis and Q fever are occupational diseases for people who work with infected animals and/or their products. In this regard, it must be remembered that the rodents captured in this study came from suburban and rural areas where people and their pets live in close contact with rodents and can acquire these pathogens (Arricau-Bouvery & Rodolakis 2005). Consequently, this study suggests that the incidence of both Q fever and toxoplasmosis, mainly in their mild forms, may have been underestimated in the Canary Islands and Cape Verde, especially in immunosuppressed patients such as pregnant women and HIV-positive people, as both pathogens can produce severe disease. For this reason, *T. gondii* and *C. burnetii* are important in sub-Saharan Africa, where HIV is a public health problem (UNAIDS 2013).

## Conclusion

The present study reveals the presence and wide distribution of *T. gondii* and *C. burnetii* in peridomestic rodents from the Canary Islands and *C. burnetii* in Cape Verde, where there were no available data about their presence in these mammals. The two species examined, *R. rattus* and* M. musculus*, presented antibodies against both pathogens with similar prevalences.

Considering the potential health impact and the economic importance of *T. gondii* and *C. burnetii* in the archipelagos studied, the results of the study could be useful for governments to improve the management of Q fever and toxoplasmosis. However, more studies are required in order to analyse the possible role of these rodent species in the transmission of both pathogens to humans and livestock in this area.
